# Innocentitis

**Published:** 1917-06

**Authors:** 


### Innocentitis.

An indictment of the whole medical profession which appears in a recent issue of the TEXAS MEDICAL JOURNAL is so ill founded, unwarranted and unjust that we cannot refrain from referring to it with indignation and regret; indignation at the lack of regard for the great harm that may be done to many clean, high-minded men totally innocent of the charges made, and sincere regret that a responsible publication would lend its pages to such a base and unmerited accusation. In all earnestness and sincerity, we wish to protest against the statement that vulgar and questionable stories are common at medical banquets or social gatherings. The writer has probably attended rather more medical meetings, conventions and conferences, with their incidental social functions, "smokers," etc., during the past twenty years, than the average physician, and he is pleased and proud to say that he never yet has heard any doctor use language to which any one could offer the least objection. The writer's experience may have been exceptional, but he does not believe so. As a matter of fact, the great majority of men have an aversion to listening to "off color" stories, especially when recited in public. Doctors offer no exception to the rule, and it is a false and wicked idea to advance that there is something in the study or practice of medicine that makes medical men particularly fond of *risque* stories. In our opinion, the reverse is the truth, and the obligations and duties of a physician's life soon fill the young practitioner with a sober sense of responsibility that rarely fails to refine and uplift him; things low, petty and sordid become distasteful and hateful. Not only does his mental and moral stature increase, but he becomes proud and jealous of the higher, nobler aims and purposes that make up his life. Men of this quality—and we can say without fear of contradiction that it describes the average physician who is imbued with the spirit of his calling—has no inclination to hear or tell stories of a vulgar nature.

As a matter purely of respect for himself and his vocation, the average physician does not want his fellow workers to associate him with stories that must be told with an apology. This is why we believe the article in the TEXAS MEDICAL JOURNAL is so mistaken in concluding that the incident on which it bases its criticism is typical of all medical meetings. Isolated instances of such conduct may occur. The medical profession has its quota of those who can be relied upon to do the wrong thing, just as every other calling has. But happily these are in the minority, and to condemn the whole profession for their mistakes is not only unfair but indicates a very imperfect knowledge of the medical profession. It seems to us that the TEXAS MEDICAL JOURNAL owes an apology to the medical profession generally—and to Texas physicians in particular—for its hasty, ill-advised and absolutely unmerited criticism.—*American Medicine.*

The TEXAS MEDICAL JOURNAL hardly expected to arouse the righteous indignation of *American Medicine* when it called attention to the rather prevalent practice of reciting risque stories at medical banquets and smokers, but is glad to have this occasion to reiterate its protest against this objectionable custom. Surely the experience of the editorial writer in *American Medicine* must have been "exceptional," as he intimates, but this JOUR-

NAL rather suspects, along with a Texas physician who called our editor's attention to the above reply, that *American Medicine* has forgotten, or else the writer has become so used to hearing the vulgar and obscene on such occasions that the worst of them appear to him like Sunday School stories. Certainly the statement that he "never yet has heard any doctor use language to which any one could offer the least objection" indicates that no language could possibly be objectionable to him, or else that his professional environment has been more saintly than falls to the lot of most men.

Perhaps physicians are not worse about this than men in other professions, but if others are worse than the physicians then the human race has our profoundest sympathy for the low plane upon which its mind feeds at banquets and smokers for "men only."

The TEXAS MEDICAL JOURNAL does not indict the entire profession, but it does protest against having the low-minded and vulgar members of the profession scatter their vile filth around banquet boards under the pretense of saying something funny or witty. It is in behalf of the decent-minded members of the profession, and their number is legion, that this protest is made. A few men of low mentality and lower moral ideals can bring discredit to a whole assembly of reputable and high-minded physicians. The better class of physicians deprecate this abominable practice, but can hardly voice their opposition at a banquet board after some member has violated all decencies of speech, and it is for this class seeking higher ideals that the TEXAS MEDICAL JOURNAL is speaking. Rest assured, dear *American Medicine,* that they do not feel aggrieved at the TEXAS MEDICAL JOURNAL for its appeal for decency. The man whose feelings are hurt is the fellow who indulges in the questionable stories.

This JOURNAL'S voice was raised in opposition to the smutty and vulgar banquet story after a number of physicians had spoken to the editor about the practice and had expressed their indignation in no uncertain terms, some of them saying that out of self-respect they had decided to quit attending medical banquets so long as so much of the speechmaking is made merely the conveyance for bad stories. Since the editorial appeared, many members of the profession have expressed their appreciation of it and have asked that the agitation for decent banquet speeches be kept up until the physician who would venture to tell a story to men that he would not tell in his own family would be hissed down.

A worse practice even than the vulgar story, though not one so frequently indulged, is the recital of professional experiences, couched in suggestive vulgarity or indecency and showing a lack of regard for professional standards or ideals.

